# *Alpinia calcarata*: potential source for the fabrication of bioactive silver nanoparticles

**DOI:** 10.1186/s40580-018-0167-9

**Published:** 2018-12-06

**Authors:** Pramila Khandel, Sushil Kumar Shahi, Deepak Kumar Soni, Ravi Kumar Yadaw, Leeladhar Kanwar

**Affiliations:** Department of Botany, Bioresource Tech Laboratory, Guru Ghasidas Vishwavidyalaya, Bilaspur, Chhattisgarh 495009 India

**Keywords:** Fabrication, Silver nanoparticles, Process optimization, Antibacterial activity, Antioxidant activity

## Abstract

In the present study silver nanoparticles fabricated by using leaf extract of *Alpinia calcarata*. We have also studied the effect of various experimental parameters viz., metal ion concentration, pH and incubation period on nanoparticle biosynthesis. Results of optimization showed that metal ion concentration of 1.5 mM, alkaline pH and incubation period of 12 h were the optimum conditions for metal nanoparticle biosynthesis. Synthesized silver nanoparticles were characterized by UV–Visible spectroscopy, Dynamic light scattering (DLS), Zeta potential analysis, Fourier transform infrared spectroscopy (FTIR), Inductively coupled plasma-optical emission spectrometry (ICP-OES), Transmission electron microscopy (TEM) and X-ray diffraction analysis (XRD). The UV–visible spectrum shows a sharp peak at 420 nm which was due to the surface plasmon resonance of the silver nanoparticles. Effect of several phytochemicals present in *A. calcarata*, on synthesis of silver nanoparticles was studied by Fourier transform infrared spectroscopy. The results indicate that the flavonoids, phytosterol, quinones and phenolic compounds present in the plant extract plays a major role in formation of silver nanoparticles in their respective ions in solution. Results of TEM and XRD analysis showed that synthesized silver nanoparticles were mostly spherical in shape with an average diameter of 27.2 ± 0.2.5 nm and highly crystalline in nature. Moreover the synthesized silver nanoparticles were also evaluated for their potential antibacterial and antioxidant activities. It showed good antibacterial activity as well as antioxidant activity. Thus the obtained result provides a scientific support that leaf extract of *A. calcarata* can be used efficiently in the production of potential bioactive silver nanoparticles with several pharmaceutical applications.
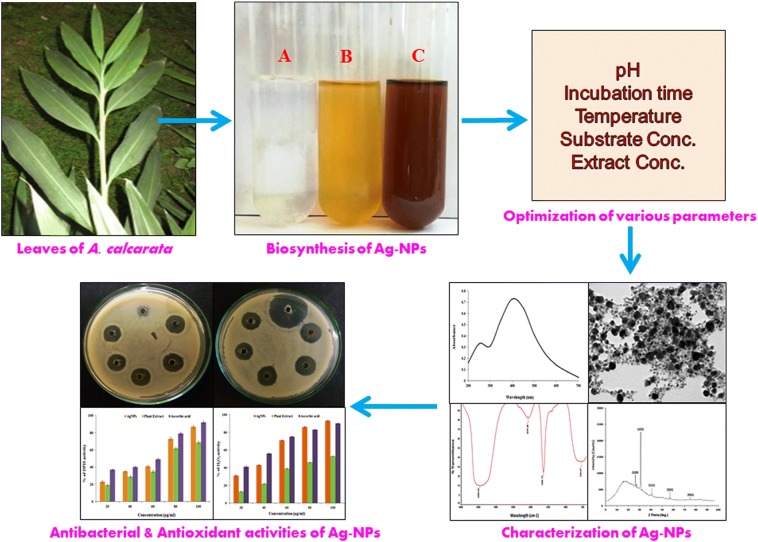

## Introduction

Fabrication methods for the synthesis of metal nanoparticles using green chemistry principle have gained significant attention in the past decades. This is mainly due to there is no requirement of toxic chemicals for synthesis protocol. The chemical synthesis approaches have drawback that during synthesis process the colloidal solution being contaminated by several by-products as a result of chemical reactions. Thus, to overcome all these problems there is a need to develop alternative green process which does not need any harmful chemicals. Considerable works have been put to develop eco-friendly approaches for the synthesis of nanoparticles. Microbes and plants are currently used for the synthesis of nanoparticles. The use of plants for the synthesis of nanoparticle is rapid, low cost, environmental benign and a single step method. Plant extract contains a variety of phytochemicals compounds such as flavonoids, terpenoids, phenols, alkaloids some plant enzymes like hydrogenases, reductases, etc. which act as a reducing and capping agent in the presence of metal salts [1].

Recently metal nanoparticles are gaining the interest of scientist for the novel methods of synthesis because they exhibit unique physicochemical properties including optical catalytic, magnetic, electronic and antimicrobial properties [2]. They have a high surface area and a high fraction of atoms. Nanomaterials such as Ag, Au, Cu, Zn, Pt and Pd have been synthesized by various methods, including biological entities using bacteria, fungi, algae and plants [3]. Among them silver (Ag) nanoparticles play a considerable role in field of medical and biological sciences due to its significant physicochemical properties. Ag nanoparticles are reported to exhibit great antibacterial, antifungal, anti-inflammatory, antioxidant and antiviral activities [4]. Nanoparticles play a crucial role in drug delivery, tissue engineering, gene delivery, artificial implants, diagnosis, imaging and sensing [5]. It has been found that a highly reactive metal nanoparticle shows excellent antimicrobial activities against bacteria [6] Silver nanoparticles have wide applications in medicines including skin creams and ointments containing silver to inhibit infection of burns and wounds and medical devices.

In the present investigation, we discuss the synthesis of bioactive Ag nanoparticles using the leaf extract of *Alpinia calcarata* as the biomaterial. *A. calcarata* is also known as Snap Ginger, which is a plant native to India, belongs to the Zingiberaceae family, as it is commonly available medicinal plant, used for synthesis of Ag nanoparticles. *A. calcarata* is a perennial herb with horizontal root stock and full leafy stem. The leaves of the plant are simple, alternative 25–35 cm in length and are 2.5–5 cm broad [7]. It is reported that the compounds isolated from the Zingiberaceae plants were found to have anticancer activity against several cell lines and also have strong anti-inflammatory and antioxidant activities [8]. Rhizome of the plant also contains various secondary metabolites majorly diterpenoids, some of them are reported as cytotoxic and can induce cell cycle arrest such as Calcarathan D, Calcarathan E etc. They also show excellent antibacterial activities against some pathogenic Gram +ve and Gram −ve bacteria. Additionally, *A. calcarata* is also used for the treatment of various diseases such as warming digestive tonic, carminative, stomachic, stimulant, expectorant and fungal infections. Plant is also used as tonic, aphrodisiac and diuretic, in the treatment of headache, lumbago diabetes, chest pain, rheumatic pains, bronchitis, dyspepsia, sore throat, impotence and diseases of kidney and liver. It is particularly considered for its efficacy in chest complains. A wide variety of chemical compounds and various bioactivities, including antimicrobial and antioxidant properties were reported in phytochemical studies from this plant. Ag nanoparticles have been synthesized using several natural products like *Crocus sativus* L. [9], *Azadirachta indica* [10], *Alysicarpus monilifer* [11], *Camellia sinensis* [12], *Glycine max* [13], *Cinnamon zeylanian* [14], *Syzygium aromaticum* [15] etc. Plants give a better platform for nanoparticle synthesis as they do not contain any toxic chemicals along with this they also provide natural stabilizing agent. Other advantages may include, use of plant extracts reduces the cost of microorganism’s isolation and culture media increases the cost ambitious viability over nanoparticle synthesis through microorganisms [16].

The aim of the present investigation was to synthesize the silver nanoparticles using leaf extract of *A. calcarata*. Phytochemical investigation of leaf extract of plant to evaluate the possible biomolecules responsible for the bioreduction and stabilization of synthesized nanoparticles. Optimization of various reaction conditions for nanoparticle synthesis such as the metal ion concentration, pH and incubation period. Furthermore characterization of synthesized silver nanoparticles by UV–Vis spectroscopy, FTIR, DLS, zeta potential measurements, ICP-OES, TEM and XRD analysis and evaluation of their potentiality for antibacterial activity against pathogenic microbial strains and antioxidant activities.

## Materials and methods

### Sample collection

Fresh leaves of *Alpinia calcarata* (Snap Ginger) were collected from campus of Department of Botany, Guru Ghasidas Vishwavidyalaya, Bilaspur, Chhattisgarh, India. Silver nitrate and other analytical grade chemicals were purchased from Hi-media laboratories, Mumbai, India. The bacterial culture of *Escherichia coli* (MTCC-44), *Pseudomonas aeruginosa* (MTCC-424) and *Staphylococcus aureus* (MTCC-96) were obtained from microbial type culture collection (MTCC), Chandigarh, India.

### Preparation of plant leaf extract

Aqueous extract of leaf of *A. calcarata* were prepared using freshly collected leaves. They were surface sterilized with running tap water followed by distilled water and finally chopped into fine pieces. About 10 g of finally incised leaf was boiled with 100 ml of distilled water for 30 min. The extract was allowed to cool than filtered with Whatman No. 1 filter paper. Filtrate was collected and stored at 4 °C for further experiment.

### Phytochemical investigation

Qualitative phytochemical analysis of *A. calcarata* was performed using the standard protocols described by Jigna and Sumitra [17] for determination of presence of several phytoconstituents like flavonoids, alkaloids, glycosides, tannins, resins, phytosterols, saponins, terpenes, quinones and phenolic compounds. The results of these tests were showed qualitatively as positive (+) or negative (**−**).

### Biosynthesis of silver nanoparticles

For biosynthesis of silver nanoparticles, 1 ml of the leaf extract was added to the 9 ml of 1 mM aqueous silver nitrate solution. The mixture was incubated in dark condition at room temperature. The visual color change in the reaction mixture from yellow to reddish brown was observed after 5 min with reference to control, which indicates the formation of silver nanoparticles. Further confirmation was done by spectrometric analysis.

### Optimization of various experimental parameters

Several experimental parameters like effect of metal ion concentration, pH and different incubation time were evaluated for biosynthesis process using *A. calcarata.* The absorbance of resultant samples was measured at 420 nm using UV–Vis spectrophotometer.

#### Effect of metal ion concentration

To study the effect of concentration of silver nitrate solution on nanoparticle synthesis, various concentration of silver nitrate (0.5–3 mM) was used. During the synthesis 9 ml of each concentration (0.5–3 mM) was taken in different test tubes. 1 ml of leaf extract was added to each of test tubes and incubated in dark condition at room temperature. Synthesis of silver nanoparticles was confirmed by UV–Vis spectrometric analysis.

#### Effect of pH

To study the effect of pH on nanoparticle biosynthesis, synthesis process was carried out at various pH ranges from (4 to 11). Silver nitrate at optimum concentration obtained by previous study was used and all other parameters for biosynthesis process were remaining the same.

#### Effect of contact time on nanoparticles biosynthesis

To study the effect of incubation period, synthesis of nanoparticle was carried at different time intervals from 0 to 16 h, absorbance of resulting solution was taken at 420 nm at various time intervals. Optimum silver nitrate concentration and pH was used from the previous experiments and other parameters were remaining the same.

### Characterization of silver nanoparticles synthesized under optimum condition

#### UV–visible spectroscopy

Initial characterization of the Ag nanoparticles was carried out using UV–visible spectroscopy. Reduction of silver ions to the silver nanoparticles was examined by measuring the surface plasmon resonance of the reaction mixture. The spectra were recorded on, Double Beam Spectrophotometer, 2203 at room temperature regulated at a resolution of 1 nm between 300 and 700 nm ranges. Optical density was taken at different wavelength ranging from 300 to 700 nm and plotted the values on a graph. Scanning was performed after reaction time ranging from 5 min to 24 h.

#### Dynamic light scattering (DLS) and Zeta potential analysis

The particle size range of the nanoparticle with their polydispersity was analyzed using zetasizer instrument (Zetasizer Nano, Malvern UK). The size of the particle was measured by the time dependent fluctuation of scattering of laser light when particles were under gone to Brownian motion. With the help of this we can also analyze the size distribution pattern and the mean size of the particle inside the sample.

For measurements of zeta potential to infer about their stability of the colloidal AgNPs was analyzed via Zeta Sizer instrument (Malvern-Nano ZS 90). The sample was poured into sample holders of the instrument and data recorded.

#### Fourier transform infrared spectroscopy (FTIR**)**

Reaction mixture was centrifuged at 12,000 rpm for 20 min to remove the biological biomass residues. Obtained pellet were redispersed in distilled water and then again centrifuge it. This step is repeated for 2–3 times. Finally samples were dried and grinded with KBr pellets and then subjected to FTIR spectroscopy measurements. The measurements were carried out on a Thermo-Nicolet-Avatar 370 instrument in the different reflectance mode at a resolution of 4 cm^−1^ in KBr pellet.

#### Inductively coupled plasma-optical emission spectrometry (ICP-OES)

The technique is mainly used for the qualitative and quantitative determination of the metals and metalloids in the biological samples. The working principle of emission spectrometry (Perkin Elmer Optima 5300 DV ICP-OES) is based on the fact that atoms or ions in an excited state tend, to revert back to the ground state and in so doing emit characteristic wavelength and intensity of that light is proportional to the concentration of that particular element in the sample solution.

#### Transmission electron microscopy (TEM)

The morphology including their shape and size were determined by transmission electron microscopy (TEM CM 200 instrument). For TEM study sample is prepared by sonicated it first for 20 min and then a drop of it was placed on copper grid and it was allowed to dry in vacuum, resulting an image is produced from the introduction of the electron transmitted through the sample.

#### X-ray diffraction pattern measurement

The X-ray diffraction pattern of biosynthesised silver nanoparticles was performed on a diffractometer (Bruker AXSD8 Advance). Synthesized silver nanoparticle solution was cast onto glass slides, and operated at a voltage of 20 mA with Cu, radiation of 1.5406 nm wavelengths. Scanning was done in the range of 2θ from 10^o^ to 90^o^. Size of synthesised nanoparticles was calculated by using Scherer’s equation,

$${\text{D }} = {\text{ k}}\uplambda /\upbeta {\mkern 1mu} {\mkern 1mu} { \cos }\uptheta$$

where D is average crystal size, k is Scherer coefficient (0.89), λ is X-ray wavelength (λ = 1.5406 nm, θ is Bragg’s angle (2θ), β is the full width at half maximum (FWHM) in radians.

### Bactericidal activity of synthesized silver nanoparticles

The Ag nanoparticles synthesized by using leaf extract was tested for dose dependent antibacterial activities by agar well diffusion method against pathogenic bacterial strains *E.* *coli*, *P. aeruginosa* (Gram −ve), and *S. aureus* (Gram +ve). In brief, the pure cultures of bacteria were subcultured on nutrient agar media (NAM). Each strain was swabbed uniformly on the individual plates. Wells of 6 mm diameter were made on nutrient agar plates by using cork borer. Various volumes of Ag nanoparticles (20, 30 and 50 µl) were added to the centre of the well. The streptomycin (1 mg/ml) and plant extract were used as a positive and negative control for the antibacterial assay. Inoculated plates were incubated at 37 °C for 24 h. After incubation, the different levels of zone of inhibition of bacteria were measured and recorded. The standard deviation was calculated using three replicates of experiments. The results of antibacterial activity were compared with control experiment.

### In vitro antioxidant activities of synthesized silver nanoparticles

#### DPPH free radical scavenging assay

The DPPH free radical scavenging activity of synthesized silver nanoparticles and plant extracts were carried out by taking absorbance at 517 nm using UV–visible spectrophotometer according to the method reported by Govindappa et al. [18]. Activity in terms of percentage was calculated using the following equation

1 $$\% {\text{ of antioxidant activity }} = \, \left( {{\text{Ac}} - {\text{As}}} \right) \, /{\text{Ac}} \times 100,$$

where Ac is the absorbance of the control sample and As is the absorbance of test sample. The results were expressed in terms of percentage of radical scavenging activity using ascorbic acid as standard.

#### Hydrogen peroxide scavenging assay

The H_2_O_2_ scavenging activity was evaluated by the method describes by Bhakya et al. [19]. Different concentration of silver nanoparticles and plant extract were mixed with 50 µl of 5 mM H_2_O_2_ solution and incubated at room temperature for 20 min. Ascorbic acid was used as standard. Absorbance was measured at 610 nm. Activity in terms of H_2_O_2_ scavenging was calculated using above mentioned (Eq. 1).

### Statistical analysis

All the experiments were performed in triplicates and the results were expressed as mean ± standard deviation. Significant levels were tested at *P *< 0.05.

## Results and discussion

In the present investigation we have demonstrated the biosynthesis of silver nanoparticles using leaf extracts of *A. calcarata.* Qualitative phytochemical analysis was performed to evaluate the phytoconstituents present in the leaf extracts. Effect of various experimental parameters on silver nanoparticle biosynthesis was also studied. Further, silver nanoparticles synthesized under optimum conditions were characterized by different analytical instruments. Bioactivity of synthesized silver nanoparticles were evaluated by antibacterial and antioxidant assay.

### Phytochemical screening

To evaluate the various phyto-constituents in the leaf extract of *A. calcarata* using different solvent extracts, a series of qualitative phytochemical tests were performed. Phytochemicals present in the different solvent extracts of *A. calcarata* are shown in Table 1 and include primarily phytosterols, phenolic compounds, quinones and flavonoids. The aqueous *A. calcarata* leaf extract consist a high amount of flavonoids and phenolic compounds suggesting that these biomolecules are the key components for synthesis of these silver nanoparticles. Flavonoids and phenolic compounds are secondary plant metabolites and many of these have been shown to contain high levels of antioxidant potentials. These phytochemicals are reported as antioxidant or free radicals scavengers, because they contain hydroxyl groups in their structure which leads to the antioxidant activities [20]. Moreover phenolic compounds are considered as effective hydrogen donors, which make them excellent antioxidants. The biomedical applications and multiple pharmaceutical activities of flavonoids and phenolic compounds have also been reported for other plant species [21]. In plants some water soluble bioorganic compounds are present which were thought to be the key components for the reduction of Ag ions to nano sized Ag nanoparticles. Previous studies also reported that biomolecules present in the leaf extract of *A. calcarata* are phenolic compounds, quinones, tannins, glycosides and flavonoids that are also considered as natural bioactive compounds [22, 23]. The biomolecules present in the leaf extract are that belongs to the phenol containing groups [24]. Ag^+^ have ability to form intermediate complexes with –OH or C=O (Hydroxyl or carboxyl groups) present in the leaf extract as a key components, which subsequently involve reduction to –COOH forms and leads to bioreduction of Ag^+^ to Ag nanoparticles [19].

**Table 1:** Phytochemical analysis of leaf extract of *Alpinia calcarata* in different solvents

Phytochemical test	Different solvents				
	Chloroform	Acetone	Methanol	Ethanol	Aqueous
Alkaloids	−	−	−	+	−
Saponins	−	−	+	−	−
Phytosterol	−	−	+	−	+
Flavonoids	+	+	−	++	++
Quinones	−	+	+	+	+
Tannins	−	−	−	+	+
Phenolic compounds	−	+	+	+	++
Glycosides	−	−	−	−	−
Resins	−	−	+	+	−

(+) fairly present; (++) highly present; (−) absent

### Biosynthesis of silver nanoparticles

**Fig. 1:**
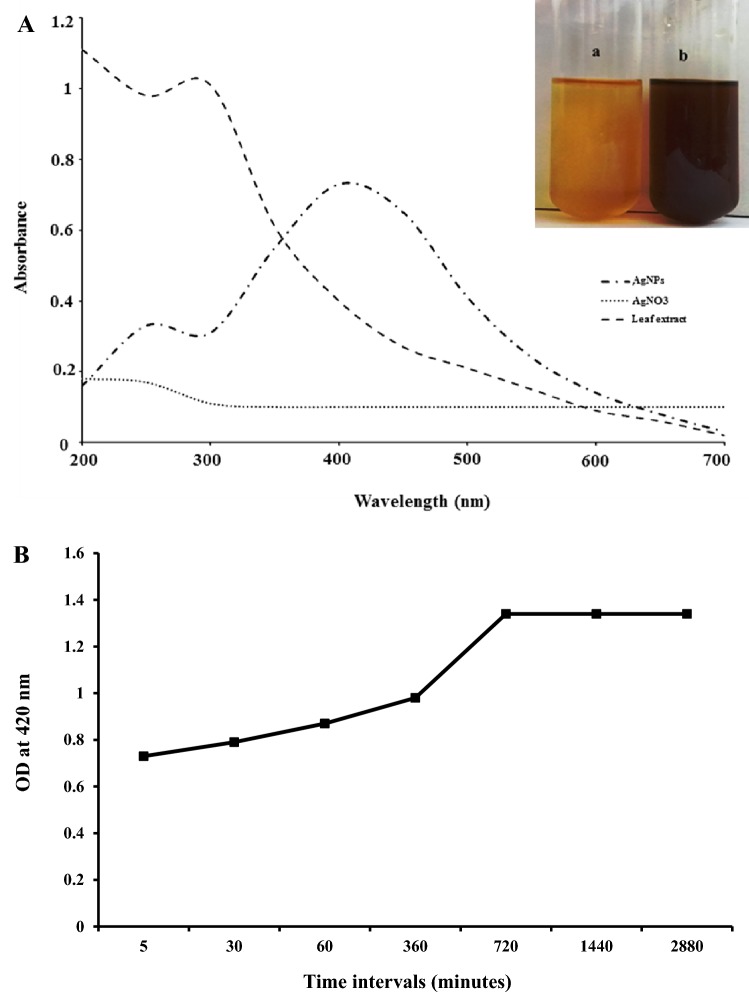
**A** UV–Visible spectrum of synthesized silver nanoparticles [inset: change in color of the solution confirming the formation of silver nanoparticles]. (a) Plant extract and (b) synthesized silver nanoparticles. **B** Color intensity at 420 nm for silver nanoparticles synthesized by *A. calcarata* leaf extract during different time intervals

In aqueous medium silver nanoparticles appears brownish red in color due to surface plasmon vibrations [2]. When the aqueous plant extract was mixed with 1 mM AgNO_3_ solution, reaction started within few minutes, and the reaction color was observed in which yellowish plant extract solution changed into reddish brown color that confirms the formation of silver nanoparticles (Inset Fig. 1A). Similar color changes were observed by Ravichandran et al. [25] when they mixed the leaf extract of *Atrocarpus altilis* with the AgNO_3_ solution. Edison et al. [26] observed the similar color changes of the plant extract *Terminalia cuneata* after 72 h of incubation at dark condition. Nanoparticles are generally characterized by their size, shape, surface area and dispersity. Change in color was mainly due to the excitation of surface plasmon resonance in metal nanoparticles [27]. The change in color is also one of the significant evidence for formation of silver nanoparticles. Our results were also corroborated with previous studies [28–30], who reported the formation of silver nanoparticles using plant extracts. Variation in the time of bioreduction of is might be due to the differences in the activities of the enzymes present in plant extract.

### Optimization of various experimental parameters

Optimization of experimental conditions is essential in order to achieve the optimum conditions for silver nanoparticles formation. The optimizing factors involved in this study were silver nitrate concentration, pH and incubation period.

#### Effect of metal ion concentration

**Fig. 2:**
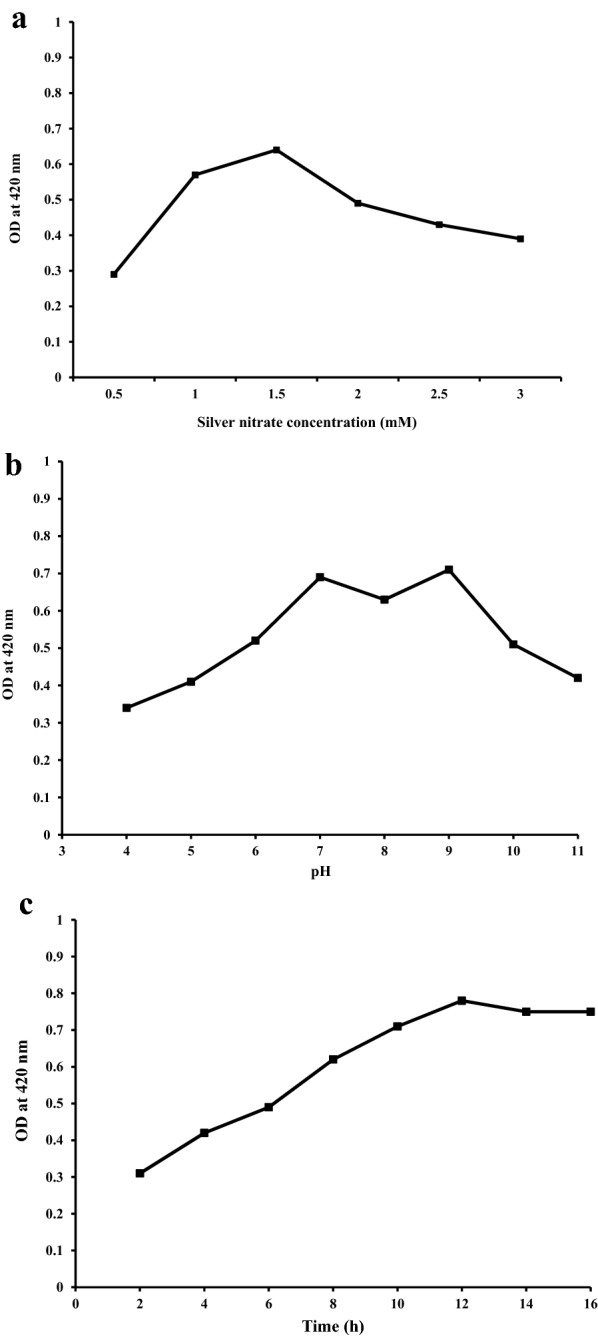
Optimization of several experimental parameters on biosynthesis of silver nanoparticles (**a**). Effect of metal ion concentration on silver nanoparticle biosynthesis (**b**). Effect of pH on silver nanoparticle biosynthesis (**c**). Effect of time (incubation period) on silver nanoparticle biosynthesis

Effect of various substrate concentrations in the reaction mixture was shown in (Fig. 2a). It is clear that they had an obvious influence on the biosynthesis of silver nanoparticles. A concentration of 1.5 mM of silver nitrate was found to be optimum concentration for biosynthesis process. Whereas other concentrations (2–3 mM) enable the synthesis of silver nanoparticles, but we have chosen the 1.5 mM concentration due to their less toxicity as compared to the other concentrations. Previous reports also suggested that higher concentration of silver nitrate produces larger particle size [31]. It was also reported that when we increase the substrate concentration above than 3 mM, they are unstable and show aggregation and precipitation at the bottom of the reaction mixture.

#### Effect of pH

To study the effect of the pH on nanoparticle biosynthesis we have selected a pH range of 4–11. Synthesized silver nanoparticles stability were determined by their corresponding absorbance at 420 nm at pH from 4 to 11 (Fig. 2b). An increase in the absorbance with increase in pH suggested that alkaline reaction condition was more appropriate for silver nanoparticle biosynthesis. However, when we increase the pH from 9 to 11, aggregation of nanoparticles was observed. Previous studies also show that optimal pH for silver nanoparticle biosynthesis has varied by plant species. Spectral responses of individual nanoparticles are related with the size and shape of the nanoparticles. It has been reported that change in pH affects the size and shape of the particles as pH has the ability to change of biomolecules that might affect their reduction as well as stability. Our results were also shows similarity with the findings of Prathna et al. [32].

#### Effect of contact time on nanoparticles biosynthesis

Figure 2c shows the effect of various incubation periods (time) on biosynthesis of silver nanoparticles. As we increase the incubation period the absorbance value was also increases up to 12 h, but thereafter they did not show any increase. In another study Pugazhendhi et al. [23] *A. calcarata* shows the formation of silver nanoparticles after 24 h of incubation.

### Characterization of synthesized silver nanoparticles

#### UV–Vis spectroscopy

UV–Vis spectroscopy analysis is used to determine the formation and stability of silver nanoparticles in the aqueous solution [23]. In aqueous suspension the size and shape of silver nanoparticle is generally recognized with UV–visible spectroscopy. The formation of silver nanoparticles was observed using UV–visible spectroscopy 200–700 nm wavelength range. In our result highest peak was observed at 420 nm (Fig. 1A), which was found to be specific for the biosynthesis of silver nanoparticles. The reaction mixture showed a single SPR band, which confirms the spherical shape of silver nanoparticles [3], which was further confirmed by TEM micrographs. The UV–Vis spectra also showed that the silver nanoparticles formed rapidly within 10 min and remained stable even after 48 h (Fig. 1B). Intense plasmon band was observed after 5 min between 400 and 430 nm, which indicates its high reducing efficiency. No change in absorbance was recorded after 48 h of incubation, confirming the complete reduction of Ag^+^ ions to silver nanoparticles. Optical absorbance spectrum of metal nanoparticles is due to SPR (surface plasmon resonance), which leads to a shift towards the red or blue ends depending upon the shape and size of the particle, surrounding medium and state of agglomeration [33]. Stability of the synthesized silver nanoparticles was studied by, measuring its intensity by UV–Vis spectrophotometer over a period of 3 months in same reaction conditions. No significant change in the absorbance was observed, which confirmed its stability over a longer period (data not shown here). Synthesis time of silver nanoparticle is quite shorter as compared to previously reported studies with plant extract [29, 34, 35].

#### Dynamic light scattering (DLS) and Zeta potential analysis

**Fig. 3:**
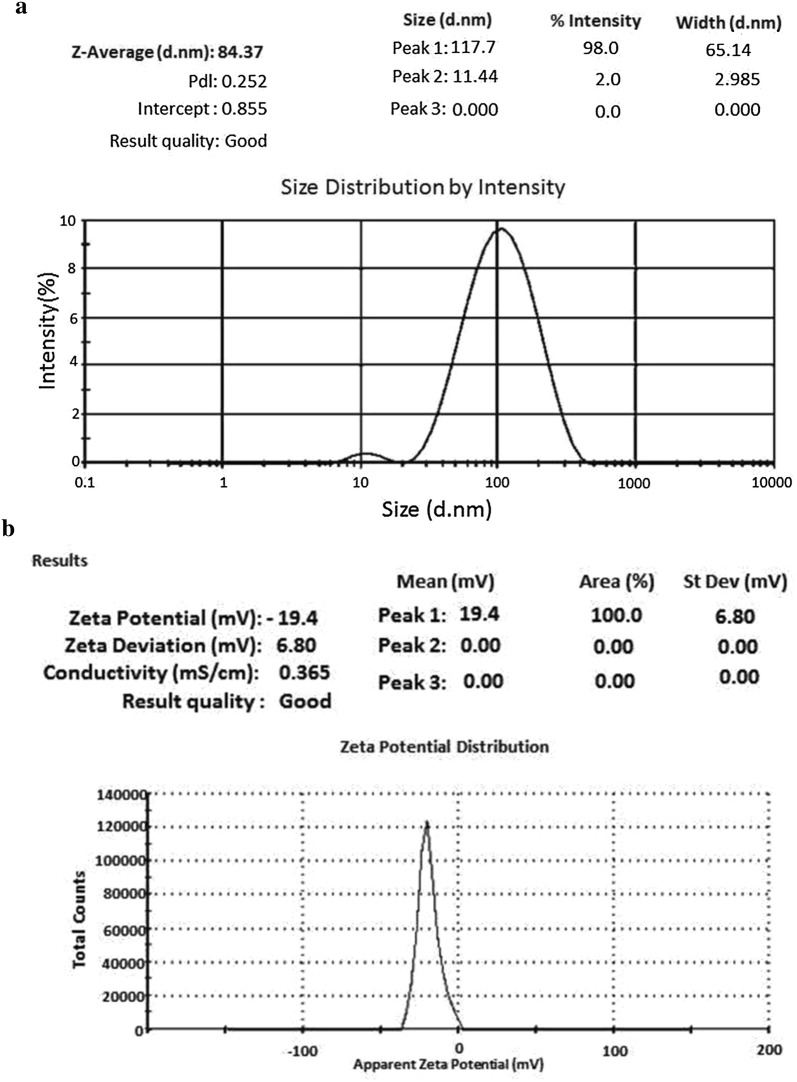
**a** DLS size distribution pattern and **b** Zeta potential analysis of synthesized silver nanoparticles

The analysis was used to analyze the size distributions and specifically measurement of monodispersity in aqueous solution. The size distribution histogram of particle size analyzer showed the Z-average diameter of 84.37 nm with polydispersity index (0.252) for biosynthesised silver nanoparticles (Fig. 3a). The size showed particle size analysis is quite larger than the size reported by TEM analysis. The variations in the results are mainly due to the process involved in the sample preparation [36]. It has been reported that the value of polydispersity index are less than 0.7 which confirms the quality of nanoparticles to be good. DLS is mainly used to analyze the quantitative size distributions as well as a more precise quantity of monodispersity in colloidal solution. Result obtained from DLS analysis shows more polydispersity and larger particle size as compared to result obtained from TEM analysis and this is mainly due to the fact that DLS analysis measured size also included the size of biomaterials that covers the surface of silver nanoparticles [19].

The surface zeta potential values of AgNPs were measured to be slightly negative and were −19.4 mV **(**Fig. 3b). Duan et al. [37] reported that AgNPs mainly exhibit negative charge. The possible cause of the negative surface charges on synthesized AgNPs is may be due to the absorption of free nitrate ions present during the reduction of AgNO_3_ [38]. The negative charge of AgNPs prevents them from agglomeration and increases their stability, as well as help to enhance their antimicrobial property. Zeta potential is mainly used for the quantification of the magnitude of charge which is a key indicator of the stability of colloidal dispersions. The magnitude of zeta potential indicates the degree of electrostatic repulsion between adjacent, similarly charged particles in dispersion. When the value for zeta potential is small, attractive force may causes the repulsion and may break and flocculate the dispersion. Nanoparticles have very small diameter and are highly energetic, this makes them highly unstable. So that particles undergo agglomeration/aggregation to stabilize themselves and also develop certain charges on their surface which contributes to their stabilization. Zeta potential has direct relation with the stability of a structure.

#### Fourier transform infrared spectroscopy (FTIR**)**

**Fig. 4:**
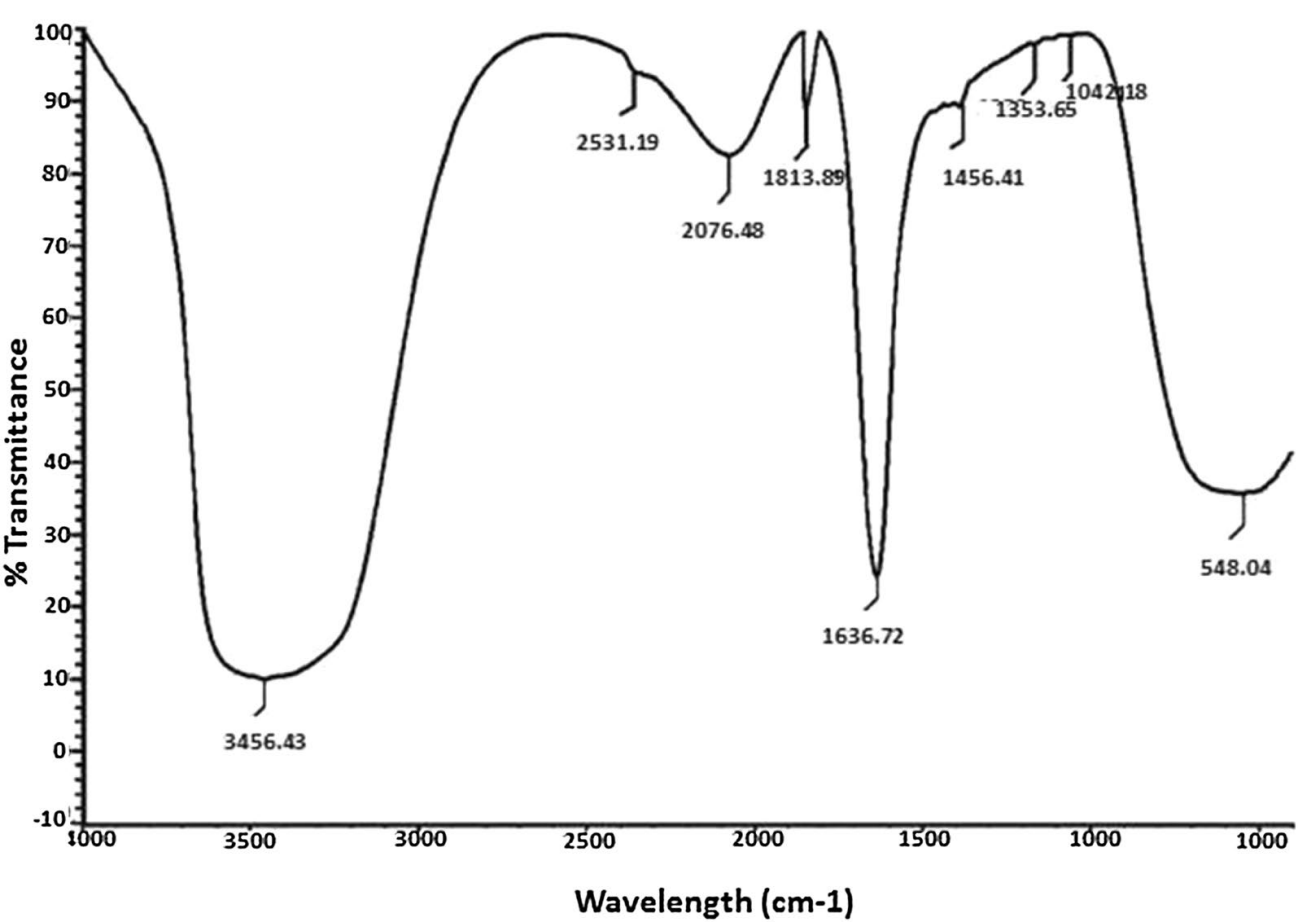
FTIR spectrum of synthesized silver nanoparticles

FTIR spectrum shows the possible biomolecules present in the leaf extract which might be involved in the reduction of silver ions. FTIR spectral measurements were studied at a resolution of 4 cm^−1^ to characterize the potential functional groups of biomolecules in the leaf extracts responsible for reduction and stabilization of bio-reduced silver nanoparticles. In our results the FTIR analysis revealed (Fig. 4) different absorption peak at 546.04 cm^−1^, 1042.18 cm^−1^, 1353.65 cm^−1^, 1456.41 cm^−1^, 1636.72 cm^−1^, 1813.89 cm^−1^, 2076.48 cm^−1^, 2531.19 cm^−1^, and at 3456.63 cm^−1^, assigned to the (C–Br), (C–O stretch), (N–O stretching), (-C-H bending) alkane, (C=C) alkene, (C=O) carbonyl stretch, (C=C) stretch, (O–H stretch) acid and (N–H) stretch or bend respectively. Sivaraman et al. [39] reported that the tannic acid which is a polyphenol and it’s a plant derived compound can be effectively reduces the silver nanoparticles. Water soluble organic compounds present in the plant materials were act as a reducing agent of silver ions to nano sized silver particles. The results indicate that the flavonoids, phytosterols, and phenolic compounds present in the plant extract play a major role in formation of silver nanoparticles in their respective ions in solution. Oxidation - reduction potential of phenolic compounds offer them to serve as a reducing agent, hydrogen donor, singlet oxygen quencher and metal ion chelators. The sharp peak at 2076.48 cm^−1^ and 3456.63 cm^−1^ attributed to the (C=C) alkyne and (N–H) amide functional groups, which indicates the presence of various aromatic and carbonyl groups of the proteins and plant metabolites present in the leaf extract that may be involved in the bioreduction process [40]. The position of these absorption bands were comparable to the phytochemicals reported in the *A. calcarata* leaf extract from Table 1. Hence, it can be concluded that the phytochemicals present in the leaf of *A. calcarata* is responsible for the reduction and stabilization of the silver nanoparticles. Similarly Vasantharaj et al. [41] reported that the phyto compounds like amides, phenols and aromatic compounds present in plant extract have strong binding efficiency with silver and also play a crucial role in reduction and stability of silver nanoparticles from Ag ions. On the basis of this we can assume that these biomolecules play an important role in the bioreduction as well as in the stabilization of metal nanoparticles [42]. FTIR results suggest that in the aqueous leaf extract water soluble phenolic groups are present which serve as a capping and stabilizing agents for silver nanoparticles, although the exact mechanism of nanoparticle formation is still unknown. Philip et al. [43] reported that flavonoids, alkaloids and polyphenols present in leaf extract of *Murraya koenigii* leaf responsible for reduction and stabilization of the synthesized gold and silver nanoparticles.

#### Inductively coupled plasma-optical emission spectrometry (ICP-OES)

The concentration of synthesized silver nanoparticles in the aqueous medium was determined by ICP-OES analysis. In this study we found that leaf extract of *A. calcarata* produces about 24.81 ppm of silver nanoparticles, per litre of culture filtrate when 1.5 mM silver nitrate was added.

#### Transmission electron microscopy (TEM)

**Fig. 5:**
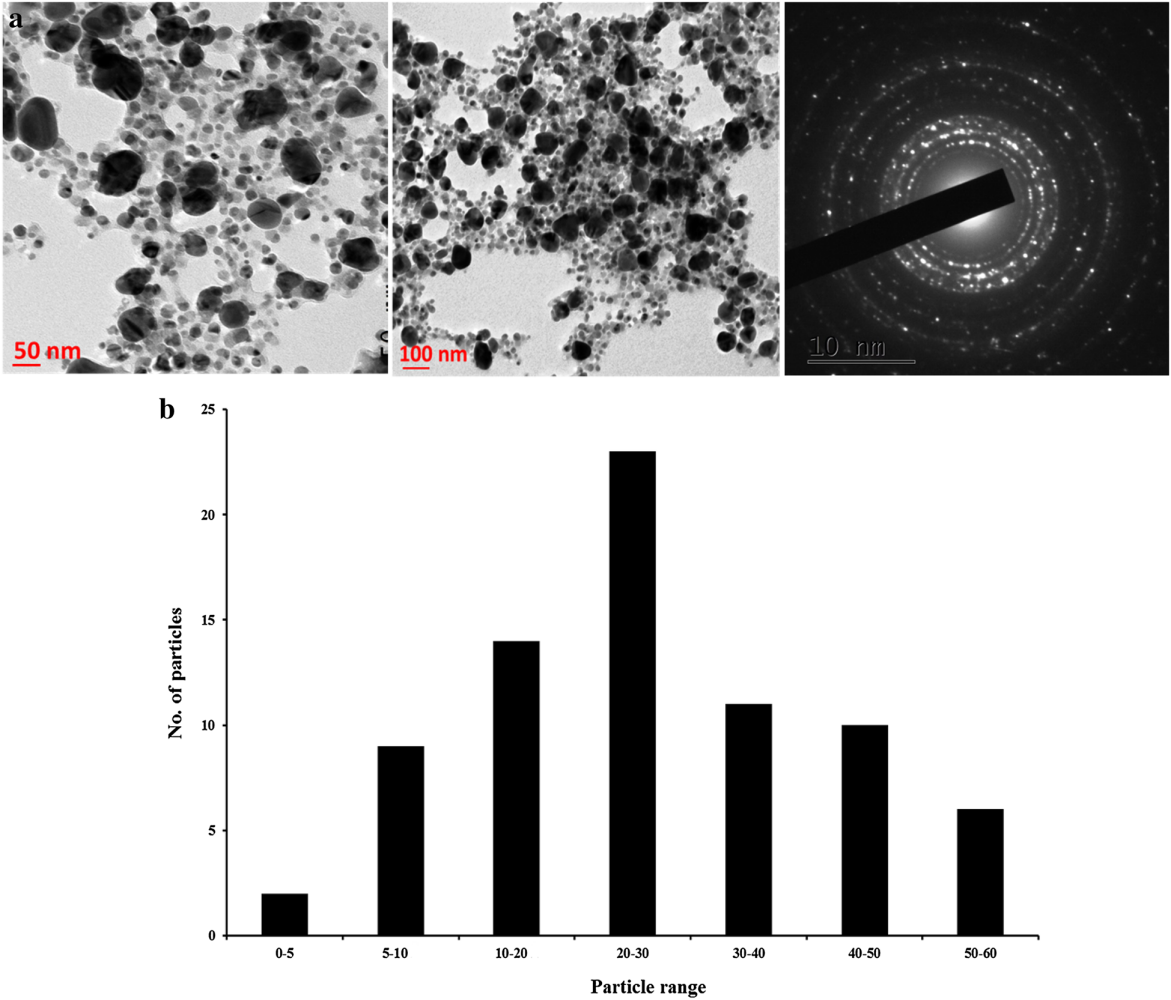
**a** TEM micrograph showing size of silver nanoparticles at 50 and100 nm scales and SAED pattern. **b** Particle size histogram of synthesized silver nanoparticles

Images of TEM analysis of silver nanoparticles revealed that well dispersed and highly crystalline silver nanoparticles were formed (Fig. 5a) and are mostly roughly spherical in shape with dark edges. Figure 5b shows the histogram pattern of synthesized silver nanoparticles. The average size of the silver nanoparticles was found to be in range of 5–60 nm with average diameter of 27.17 nm. The SAED (selected area electron diffraction) pattern of synthesized nanoparticles was also shown in the inset of (Fig. 5a). A circular ring was observed which shows the crystalline nature of the silver nanoparticles. The size and shape of the nanoparticles are important as their applications are dependent on their size and shape. Similar results were also obtained by Bar et al. [44] who synthesized the spherical shape silver nano crystals with average size in between range of 15–50 nm using latex of *Jatropha curcas* shape. In TEM micrograph it was also found that the edged of the synthesized silver nanoparticles were lighter than the centre, suggesting the presence of biomolecules like protein, polyphenols and flavonoids in leaf extract, that act as a capping agent and prevent their aggregation [23]. This type of inherent capping offers the additional benefits of the stability in the green chemistry synthesis [45]. Our results were also in agreements with the earlier reports [46–48].

#### X-ray diffraction analysis (XRD)

**Fig. 6:**
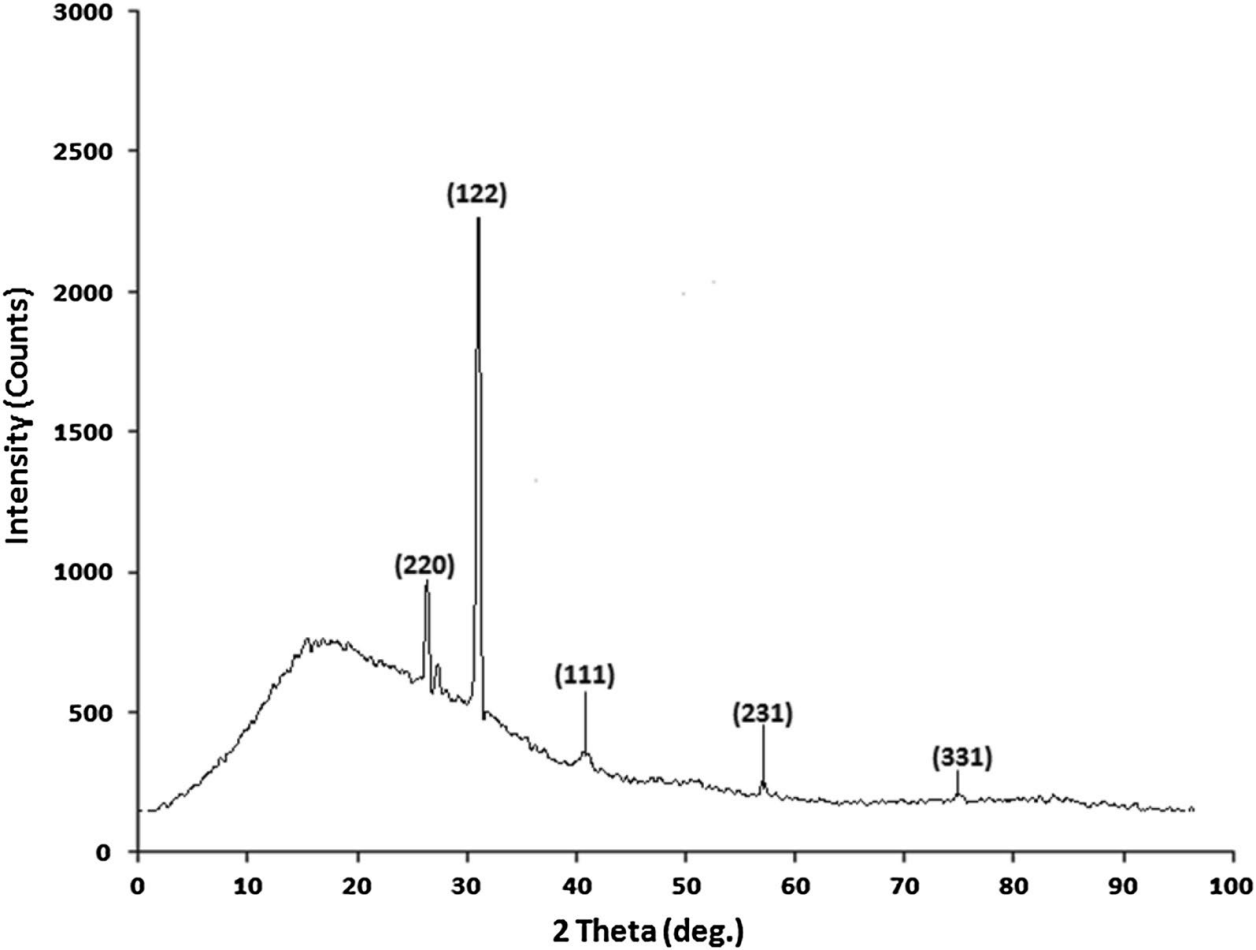
XRD pattern of the synthesized silver nanoparticles from *A. calcarata*

XRD is mainly used for the phase identification and characterization of crystalline nature of synthesized silver nanoparticles. XRD pattern of synthesized silver nanoparticles are shown in Fig. 6. Results of XRD analysis indicate that the structure of silver nanoparticles is face-centred cubic (FCC). Additionally, in our results the XRD peaks at 2θ of 28.17°, 33.31°, 44.12°, 54.12° and 77.21° could be attributed to the (220), (122), (111), (231) and (331) crystallographic planes. Thus, from the XRD analysis results it was clear that synthesized silver nanoparticles were crystalline in nature. Similar results were reported for *Ziziphora tenuior* [49], *Citrus limon, Citrus sinensis* and *Citrus reticulate* [50] and *Abelmoschus esculentus* [51]. The XRD patterns showed here are consistent with earlier reports [52–54]. Sharp peaks of XRD profile clearly indicate the cubic crystalline nature of the synthesized silver nanoparticles. Similarly Yoon et al. [54] carried out a research and they also found the peaks at 28^o^ and 32^o^ in XRD diffractogram of fungal based silver nanoparticles.

### Antibacterial activity of synthesized silver nanoparticles

**Fig. 7:**
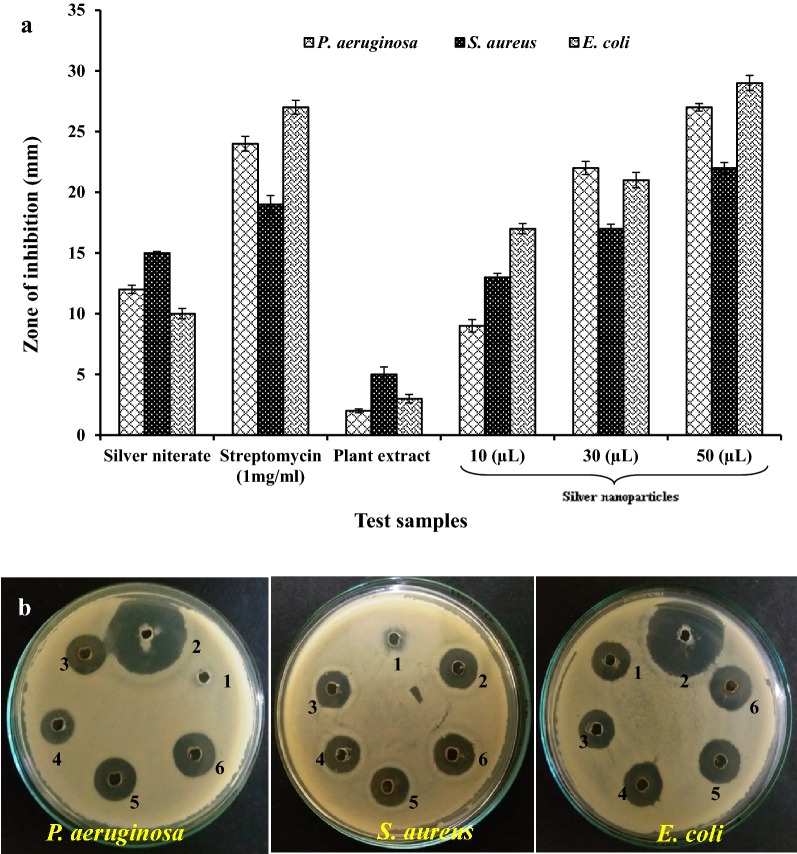
**a** Antibacterial activity of silver nanoparticles against various pathogenic bacterial strains. **b** Zone of inhibition (1) Plant extract; (2) Streptomycin; (3) Silver nitrate; (4) 10 µl silver nanoparticles; (5) 30 µl silver nanoparticles; (6) 50 µl silver nanoparticles

The susceptibility of pathogenic bacteria to silver nanoparticles showed in Table 2. Zone of inhibition of standard antibiotics like streptomycin, plant extract and various volumes of silver nanoparticles were showed in Fig. 7a, b. Volumes of silver nanoparticles vary viz, 10 µl, 30 µl and 50 µl. The results showed that the silver nanoparticles had efficient antibacterial activity against Gram-positive and Gram-negative bacteria. The leaf extract of *A. calcarata* showed significantly less activity against all the test pathogens as compare to the silver nanoparticles synthesized from leaf extract. This indicates that the biological activity of leaf extract of *A. calcarata* increased when amended with silver, which means the silver nanoparticle produced with *A. calcarata* showed good antimicrobial activity. Although silver nitrate showed a moderate activity against all the test pathogens, but due to its high toxicity against human cells, its effect was found to be insignificant. Interestingly in our results synthesized silver nanoparticles showed maximum antibacterial activity against *P. aruginosa* and *E. coli.* (Gram −ve bacteria) as compare to *S. aurueus* (Gram +ve bacteria). The result might be due to the differences in cell wall structure of Gram +ve and Gram −ve bacteria. According to Chaloupka et al. [55] the Gram +ve bacteria consist a thick layer of peptidoglycan which consist linear polysaccharide chain cross linked by short peptides, so that forming a rigid cellular structure and thus leads to difficult penetration of the silver nanoparticles. While increasing the concentration of silver nanoparticles, zone of inhibition also increases. Control shows no zone of inhibition, which indicates that the antibacterial properties is due to synthesized silver nanoparticles. Lok et al. [56] reported that bacterial cell membrane and plasma were damaged by silver nanoparticles which lead to exhaustion of the intracellular adenosine triphosphate. Elgorban et al. [57] were also studied the antibacterial effect of silver nanoparticles was depend on shape and size. Nanoparticles having size within range of 1–10 nm can attach to the cell surface and effectively damage the permeability and respiratory functions [58]. Though the antimicrobial activity by silver nanoparticle is very prominent, but their mode of action is still debatable. Sondi and Sondi [59] proposed that silver nanoparticles has the ability to attach with the bacterial cell membranes that causes change in bacterial cell membrane structure and leads to the formation of ‘pits’ where they accumulate. In another report it was supposed that silver nanoparticles releases silver ions that interact with the thiol groups of enzymes and leads to the inactivation of most of the respiratory chain enzymes due to the formation of reactive oxygen species (ROS) which causes the apoptosis of bacterial cell [60–63].

**Table 2:** Antibacterial activity of synthesized silver nanoparticles

Bacterial strains	Zone of inhibition (mean ± SD in mm)					
	AgNO_3_ (1 mM)	Streptomycin (1 mg/ml)	Plant extract (50 µl)	Synthesized AgNPs		
				10 µl	30 µl	50 µl
*E. coli*	12.17 ± 0.34	24.12 ± 0.61	2.87 ± 0.16	9.13 ± 0.52	22.13 ± 0.54	27.70 ± 0.31
*S. aureus*	15.06 ± 0.12	19.54 ± 0.73	5.38 ± 0.61	13.41 ± 0.33	17.52 ± 0.37	22.43 ± 0.45
*P. aeruginosa*	10.11 ± 0.43	27.42 ± 0.57	3.67 ± 0.35	17.09 ± 0.42	21.17 ± 0.63	29.14 ± 0.63

*SD* standard deviation

A number of reports available that shows that silver have been permanently utilized to treat or inhibit a wide range of disease caused by both Gram positive and Gram negative bacteria [64]. Results from previous studies also support the antibacterial activity of silver nanoparticles [65]. No zone of inhibition was shown in case of control. Recently nanoparticles has been used as an interesting alternative method to antibiotics and supposed to have a high potential in curing several bacterial diseases in human. In recent years silver nanoparticles have attracted significant attention especially in antimicrobial activity [66, 67].

Similar study was carried out by Paszek et al. [68], during their study they reported that the high antibacterial activity of silver nanoparticles is due to the release of silver cations from silver nanoparticles that act as reservoirs for the Ag^+^ bactericidal agent. Silver nanoparticles synthesized from various plant extracts were reported for potent bactericidal activity [69–71].

Results of this study strongly recommended that silver nanoparticle synthesized from *A. calcarata* exhibit potential antibacterial activity against disease causing pathogenic strains of bacteria and hence can be used as an antibiotic or may be used for the development of nano based antibacterial formulations.

### Antioxidant activities of synthesized silver nanoparticles

**Fig. 8:**
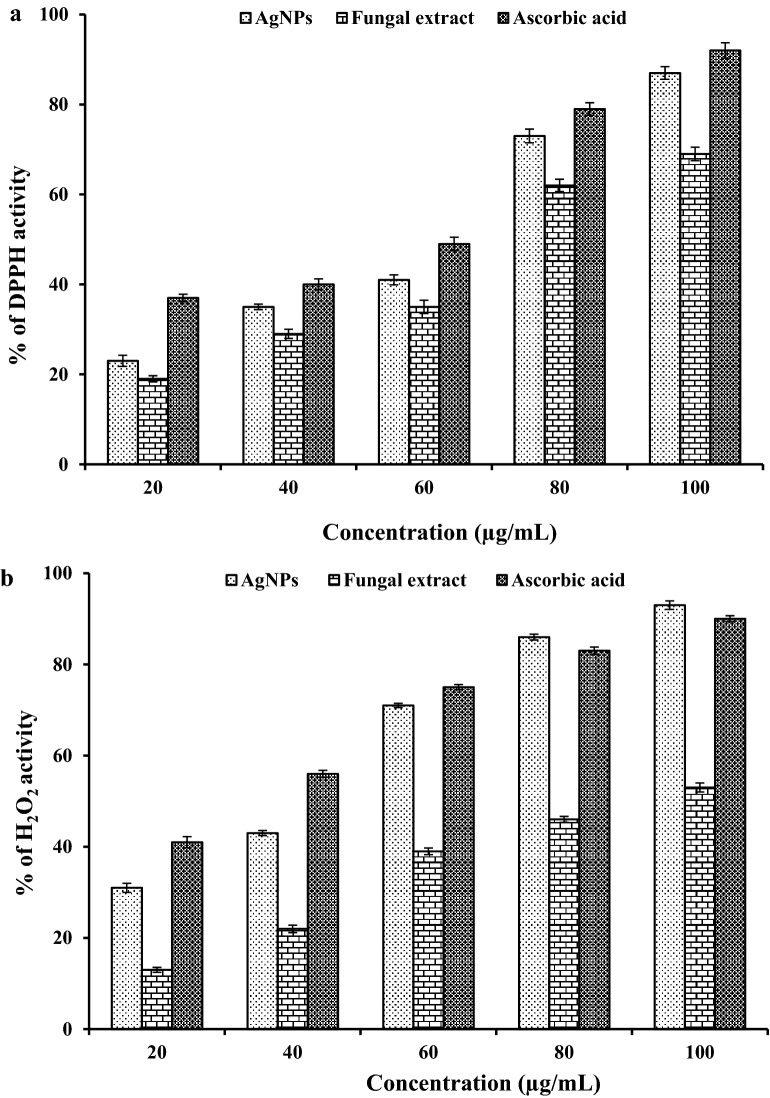
Antioxidant activities of synthesized silver nanoparticles. **a** DPPH free radical scavenging activity. **b** H_2_O_2_ scavenging activity

DPPH is a stable free radical at room temperature and accepts an electron or hydrogen radical to form stable diamagnetic molecules [71, 72]. DPPH free radical scavenging activity has been used extensively for determining antioxidants such as polyphenols. The free radical scavenging activity of silver nanoparticles was assessed by observing the visual color changes from purple to yellow after reduction, while the control does not show any color changes. As compared to the standard ascorbic acid, synthesized silver nanoparticles shows effective DPPH scavenging activity (Fig. 8a). It has been also revealed that the antioxidant potentials of silver nanoparticles increase in a dose dependent manner. Instead of this silver nanoparticle also shows more inhibition with 90% scavenging activity of DPPH. Our results were corroborated with Ragini et al. [73], who reported the free radical scavenging activity of *Shorea tumbuggaia*, and found 79.50% scavenging activity at a 100 µg/ml concentration. Various plant extract exhibit efficient antioxidant activities due to their phytochemicals, majorly incudes phenols and flavonoids [74].

The H_2_O_2_ scavenging activity of silver nanoparticles was quantitatively analyzed by using spectrophotometer and is shown in (Fig. 8b). As we know that hydroxyl radical is a highly reactive free radical formed in biological system and has been used as a highly damaging species in free radical pathology, having ability to damage a wide range of molecules like proteins, DNA, lipids etc. [75]. Results of present study showed that silver nanoparticles exhibited potential reducing power as compare to ascorbic acid. It has been reported that in the presence of H_2_O_2_ silver nanoparticles can hydroxyl radicals (reactive oxygen species). The free radical scavenging activity of silver nanoparticles are might be due to the presence of several phyto-constituents that have ability to donate the hydrogen atom in their OH groups. Results also resembles with earlier reports on antioxidant activity of leaf extract of *Erythrina suberosa (Roxb.)* [71]. The result obtained from this experiment also recommends the applications of silver nanoparticles as natural antioxidant agent for several health preservation against various oxidative stresses related with degenerative diseases.

## Conclusions

The present study revealed the rapid fabrication of silver nanoparticles using leaf extract of *A. calcarata.* Silver nanoparticles formation was achieved within 10 min, which was confirmed by UV–Vis spectroscopy analysis that showed a sharp peak at 420 nm confirming the formation of silver nanoparticles. The effects of various experimental parameters played a major role in the biosynthesis and size control of the particles. FTIR results suggested that the biomolecules like the secondary metabolites present in the plant leaves are responsible for the reduction and stabilization of nanoparticles. TEM micrograph of synthesized silver nanoparticles showed spherical shaped nanoparticles with average particle size 27 nm. DLS analysis showed that synthesized nanoparticles were well dispersed. XRD analysis concluded that synthesized nanoparticles were highly crystalline in nature. Antibacterial and antioxidant studies revealed that the synthesized Ag nanoparticles have potential bactericidal activities against all the selected pathogenic strains of bacteria and they also show efficient free radical scavenging activity. Hence this green chemistry principle towards the synthesis of silver nanoparticles have several benefits like rapid, convenient, facile and ease to scale up etc. Future prospects may include the formulation of nanomedicines against several human and veterinary pathogens by using such plant extracts and to develop studies in the interface between biology and material structural science.
